# Extraction, Purification, Structural Characteristics, Biological Activity and Application of Polysaccharides from *Portulaca oleracea* L. (Purslane): A Review

**DOI:** 10.3390/molecules28124813

**Published:** 2023-06-16

**Authors:** Meng Wang, Caijiao Li, Jiaye Li, Wenjing Hu, Aiqi Yu, Haipeng Tang, Jiayan Li, Haixue Kuang, Huijie Zhang

**Affiliations:** Key Laboratory of Basic and Application Research of Beiyao, Heilongjiang University of Chinese Medicine, Ministry of Education, The First Affiliated Hospital of Heilongjiang University of Chinese Medicine, Harbin 150040, China

**Keywords:** *Portulaca oleracea* L., polysaccharide, structural characteristic, biological activity, application

## Abstract

*Portulaca oleracea* L. (purslane) is a widely distributed plant with a long history of cultivation and consumption. Notably, polysaccharides obtained from purslane exhibit surprising and satisfactory biological activities, which explain the various benefits of purslane on human health, including anti-inflammatory, antidiabetic, antitumor, antifatigue, antiviral and immunomodulatory effects. This article systematically reviews the extraction and purification methods, chemical structure, chemical modification, biological activity and other aspects of polysaccharides from purslane collected in the Chinese Pharmacopoeia, Flora of China, Web of Science, PubMed, Baidu Scholar, Google Scholar and CNKI databases in the last 14 years, using the keywords “*Portulaca oleracea* L. polysaccharides” and “purslane polysaccharides”. The application of purslane polysaccharides in different fields is also summarized, and its application prospects are also discussed. This paper provides an updated and deeper understanding of purslane polysaccharides, which will provide useful guidance for the further optimization of polysaccharide structures and the development of purslane polysaccharides as a novel functional material, as well as a theoretical basis for its further research and application in human health and manufacturing development.

## 1. Introduction

**Figure 1:**
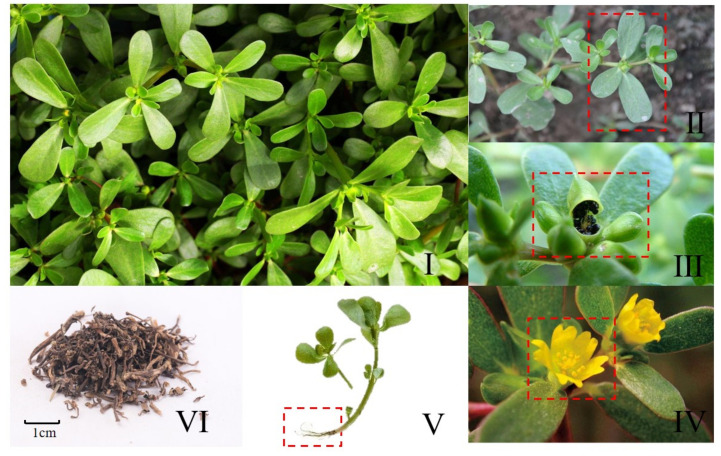
Botanical characteristics of purslane: (**I**) the plants of purslane; (**II**) the leaves of purslane; (**III**) the fruits and seeds of purslane; (**IV**) the flowers of purslane; (**V**) the roots of purslane; (**VI**) dried purslane.

*Portulaca oleracea* L. (purslane) belongs to the *Portulaceae* family and is an annual herbaceous plant. It originated in India, and has been distributed in multiple regions around the world, mainly in temperate and tropical regions such as Asia, Africa, Europe, etc. [1,2]. In different countries and language systems, the name purslane is different: it is called “rigla” in Egypt, “pigweed” in England, “pourpier” in France and “Ma-Chi-Xian” in China (Figure 1). In addition, some of its aliases can reflect its properties. In Brazil, it is called “unconventional edible plant”, which means it does not belong to a specific region or country and can even be consumed by most of the world’s population. In China, it is known as “vegetable for long life” due to its multiple benefits to the human body. Purslane is rich in nutrients such as malic acid, glucose, calcium, phosphorus and iron, as well as carotene and vitamins B, C and E. Regular consumption of purslane not only supplements the body with many nutrients, but also enhances immunity, lowers blood sugar and blood lipids and has a positive effect on protecting the eyes [3]. There are various ways to eat purslane; it is suitable for both fresh and dry food. It can be cold-mixed, stir-fried, or boiled in soup. It tastes delicious and refreshing, so it also has the effect of promoting appetite [4].

Purslane is not only a green, safe, nutritious and edible vegetable, but also a good medicine, with various health-promoting effects on the human body. In folk medicine, purslane is commonly used to treat various diseases, and has satisfying effects [5]. For example, in Afghanistan, purslane is used to prevent diarrhea and throat infections. Sri Lanka uses purslane to treat ulcers, wounds, burns and skin diseases. In Dominica and the West Indies, it is used to treat intestinal worms. In addition, purslane is known as a “natural antibiotic”, because it has a significant inhibitory effect on *Typhoid bacillus*, *Escherichia coli* and *Dysentery bacillus*. Officially, the 2020 edition of the Chinese Pharmacopoeia contains purslane, which is clinically used for eczema, snakebites, blood in stool, hemorrhoids, etc. Moreover, it is also included in the “Affinal Drugs and Diet” Catalogue issued by the Chinese Health Commission, indicating that its health benefits have been recognized by national authoritative departments. This also means that it has dual properties as a natural plant and can be safely eaten [6,7]. Therefore, purslane has attracted the attention of more and more researchers, as well as consumers who focus on dietary balance and physical health. Purslane is appearing more frequently on people’s dining tables as a healthy food source.

Its extensive clinical effects are closely related to its chemical composition. According to existing research and reports, purslane contains various chemical components, including polysaccharides, alkaloids, flavonoids and terpenoids [8,9]. Among them, polysaccharides, as an important macromolecule, have been increasingly studied due to their natural origin, safety and low toxicity. At present, multiple structurally diverse polysaccharides from purslane have been obtained through different extraction and purification methods. They have been proven to have health benefits for the human body, including anti-inflammatory, antidiabetic, antitumor, antiviral, anti-lead poisoning, antifatigue and immunomodulatory effects [10]. This gives it broad application prospects in fields such as food, pharmaceuticals, cosmetics and animal husbandry.

Through searching the existing literature on purslane, we found that there are some review articles on purslane and its bioactive components, but the main focus is on the small molecular compounds of purslane and their pharmacological effects, and there is a lack of comprehensive reviews on the macromolecular polysaccharides from purslane. Therefore, this review systematically combs the major research on the extraction and purification methods of polysaccharides from purslane in recent years, and reviews their chemical structure, structural modification, health benefits and applications in different fields of purslane polysaccharides. It is hoped that more researchers will pay attention to its significant and reliable biological activity, to better utilize it. In addition, this will also provide a theoretical basis for further research and development of functional products related to purslane polysaccharides, and contribute to further diversification of the purslane polysaccharide industry.

## 2. Extraction and Purification Methods of Purslane Polysaccharides

For the study of purslane polysaccharides, the extraction of them is a very important step. Before extraction, purslane powder was degreased to remove the lipophaphilic components using petroleum ether or 80% ethanol. Then, after the degreased purslane was dried, the polysaccharide was extracted. When the purslane polysaccharides were extracted by hot water extraction (HWE), the extraction temperature was from 75 °C to 100 °C, the extraction time was from 2 h to 15 h, and extraction was performed between 1 and 3 times. HWE is the most commonly used method for the extraction of purslane polysaccharides, which is simple, safe and does not cause pollution. However, this method is time-consuming, and the yield of polysaccharides is low [11]. Based on these limitations, some novel and efficient extraction methods have been introduced recently. By using the cavitation effect of ultrasonic waves, ultrasonic-assisted extraction (UAE) accelerates the cell wall rupture to release and diffuse the polysaccharide from the cell, thereby improving the extraction rate of polysaccharides. Compared with HWE, UAE shortens the extraction time from 120 min to 53.08 min and increases the extraction rate from 4.84% to 13.55%. Microwave-assisted extraction (MAE) is a method of extracting bioactive components by microwave energy. Here, the entire volume of the extraction mixture is rapidly heated, which shortens the extraction time and improves the extraction yield. Moreover, neither its structure nor the biological activity is affected. When MAE method was used to extract purslane polysaccharides, the extraction time was 10 min, the extraction rate was 13.87%. EAE is considered to be an extraction method with mild conditions, high recovery and environmental protection. When extracting purslane polysaccharides, 0.15 g/kg pectinase is usually used, which can hydrolyze plant cell wall and dissolve purslane polysaccharides, and the yield is 4.22 g/kg. Compared with single-enzyme extraction, double-enzyme extraction has shorter extraction time and higher extraction rate. Studies have shown that when the dosage of cellulase and pectinase is 1.5% and 2.0%, the enzymolysis time is 100 min, the temperature is 50 °C and the ratio of solid to liquid is 1:25, the yield of purslane polysaccharides is 19.83 mg/g. Moreover, ultrahigh-pressure (UHP) extraction means that the solvent can penetrate into the raw material under the condition of ultrahigh pressure, so that the active component is dissolved in the solvent, which has the advantages of fast extraction speed, high efficiency, low energy consumption, and simple operation. The extraction time of purslane polysaccharides was 5 min, and the extraction rate was 22.21% by UHP extraction. In addition to the single extraction method, several methods can be combined to synthesize the advantages of each extraction method to extract purslane polysaccharides more completely. Ultrasonic-assisted enzymatic extraction (UAEE) is a complex extraction method combining ultrasound and enzymes; the extraction rate increased from 19.72 mg/g to 21.23 mg/g, compared with the single UAE. Different extraction methods have advantages and disadvantages. According to the actual situation, the appropriate extraction method was selected to better study the purslane polysaccharides.

**Figure 2:**
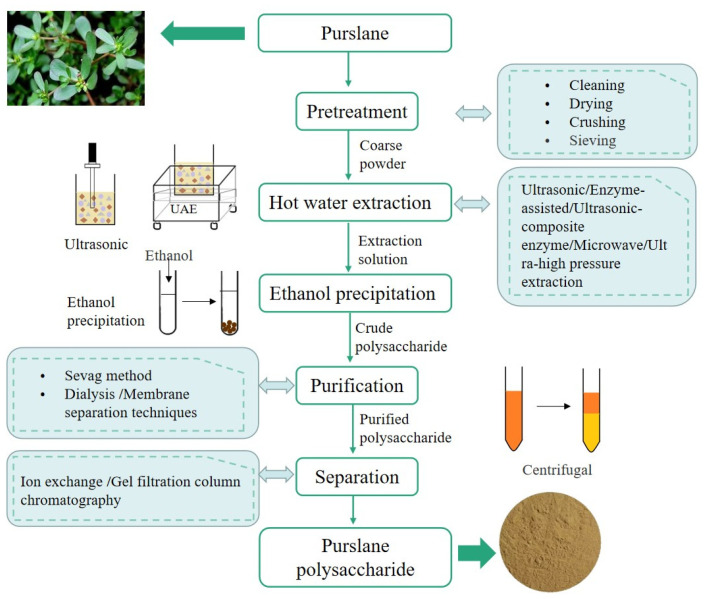
Flow chart of purslane polysaccharide extraction and purification.

The crude purslane polysaccharides were obtained after water extraction and alcohol precipitation [11]. The crude polysaccharides often contained impurities such as protein and inorganic salt, which needed further purification. The Sevag method is the mildest and most commonly used method for the deproteinization of polysaccharides, based on the principle that proteins are denatured and precipitated in organic solvents and are insoluble in water. Dialysis and membrane separation techniques are the commonly used methods for removing inorganic salts from purslane polysaccharides in laboratory [12]. In addition, lipids and pigments can be removed by soaking in anhydrous ethers or washing with acetone and tetrachloromethane. After removing impurities, the crude purslane polysaccharides were further purified and separated by ion exchange chromatography and gel filtration chromatography [13,14]. Finally, the polysaccharide solution was dried and purified purslane polysaccharides were obtained, which was stored at −20 °C. The flow chart of purslane polysaccharide extraction and purification is shown in Figure 2.

In conclusion, obtaining polysaccharides from purslane requires the steps of extraction, separation and purification. Each step of treatment may affect its yield, structure and activity. Therefore, it is important to carefully select suitable, reasonable, safe and effective extraction and purification methods.

## 3. Structural Characteristics of Purslane

At present, there are ten kinds of polysaccharides isolated from purslane, which have different molecular weights, compositions and structures. The polysaccharides from purslane and their names, molecular weights, monosaccharide compositions and references are listed in Table 1.

### 3.1. Molecular Weight

The average molecular weight (Mw) of purslane polysaccharides was different due to different extraction, purification and analysis methods. The average molecular weight of purslane polysaccharides was determined by high-performance liquid chromatography (HPLC), high-performance gel permeation chromatography (HPGPC) and gas chromatography (GC). The average molecular weight distribution of purslane polysaccharides was very wide, ranging from 8.3 kDa to 15,500 kDa. Zhao et al. obtained polysaccharides (POL-P3b) from purslane through separation and purification. POL-P3b was identified as a homogeneous polysaccharide component with an average molecular weight of 253.6 Da. A special component, galacturonic acid (designated as POPW-HG), was purified and isolated from purslane polysaccharides, and its average molecular weight was 41.2 kDa. In addition, the molecular weights of polysaccharides neutral polysaccharide (RN), acidic polysaccharide (RA) and pectin polysaccharide (RP) obtained from purslane were 8.3 kDa, 58 kDa and 87 kDa, respectively. In particular, there were three different molecular weight distribution ranges of CPOP. The main molecular weight distribution of CPOP was 7.3 kDa, and the two minor molecular weight distributions were 11.9 kDa and 93 k Da.

### 3.2. Monosaccharide Composition

The bioactivity of polysaccharides is closely related to its structure, especially the composition and proportion of monosaccharides. The monosaccharides of purslane polysaccharides were determined by infrared spectroscopy (IR), GC and HPLC. The results showed that the purslane polysaccharides were mainly composed of mannose (Man), rhamnose (Rha), glucuronic acid (GlcA), galacturonic acid (GalA), glucose (Glc), galactose (Gal) and arabinose (Ara) in different molar ratios. Three different types of polysaccharides were extracted from purslane: neutral polysaccharide (RN), which was mainly composed of Glc, Man, Ara and Gal with molar ratio of 0.1, 38.8, 13.7, and 5.3; acidic polysaccharide (RA), mainly composed of Ara, Gal, Rha, Xyl and GlcA processed with a molar ratio of 23.3:67.0:2.8:2.9:4.1; and a pectin polysaccharide (RP), composed of GalA, Gal, GlcA, Ara and Rha with a molar ratio of 67.8:11.3:10.6:5.8:4.3 [19,20,29]. In addition, the polysaccharide POPW-HG isolated from purslane is a homogeneous polysaccharide consisting of 95% galacturonic acid [21]. The homogenous polysaccharide POL-P3b obtained from purslane was composed of Glc and Gal with a molar ratio of 0.75:1.00.

### 3.3. Chemical Structures

In addition to monosaccharide composition and molecular weight, the chemical structure of purslane polysaccharides has been studied extensively. The structure and conformational characteristics of polysaccharides from purslane were characterized by IR, GC and nuclear magnetic resonance (NMR) spectroscopy [33,34]. Tao et al. studied the basic characteristics of polysaccharides from purslane by Fourier transform infrared spectroscopy (FI-IR). The results showed that there were hydroxyl groups, C-O and uronic acid in the structure of purslane polysaccharides. Zhao et al. conducted a basic study on the structure of POL-P3b through IR. Analysis showed that the purified POL-P3b structure had hydroxyl groups and β-glucoside bonds. Dong et al. isolated three polysaccharides from purslane, which were RN, RA and RP. RN is an arabinoglucomannan-type polysaccharide. Methylation analysis showed that RN contained 1,4-linked mannopyranosyl (Manp), 1,4-linked glucopyranosyl (Glcp) and 1,2,4-linked Glcp residues with small amounts of 1,4,6-linked Manp, 1,4,6-linked Glcp and terminal-linked galactopyranosyl (Galp) residues. In addition, arabinofuranosyl (Araf) residue was suggested to be mainly present as nonreducing terminal-linked residues. Methylation analysis showed that RA is a typical type II arabinogalactan (AGII), which consists of an *α*-1,3-linked *β*-d-Galp main chain, partially substituted at C-6 by 1,6-linked *β*-d-Galp side chains. Moreover, GlcA was present in RA in the forms of terminal-linked and 1,4-linked residues. NMR analysis suggested that RP might be a galacturonan consisting mainly of α-1,4-linked GalA residues that are highly methylated and partially acetylated. According to the reported method, the methyl esterification degree (DM) of RP was estimated to be 73.2%, indicating that RP is a highly methylated pectin polysaccharide. In addition, Selina Mawunyo Ayivi-tosuh et al. purified homolacaronic acid (POPW-HG) from purslane polysaccharides and studied it deeply. Detection by methylation analysis and GC indicated that POPW-HG consisted of only 1,4-Gal (reduced 1,4-GalA). FI-IR analysis showed that there were hydroxyl, uronic acid and pyranose rings in POPW-HG. ^1^H and ^13^C NMR analysis showed that there was no signal of GalAp methylation and acetylation, indicating that POPWHG is an unesterified homogalacturonic acid. The results show that POPW-HG is a linear and non-esterified homogalacturonan. Homogalacturonan consists of an α-1-4-d-GalAp residue unit, linear HG and branched HG. Linear HG is an unsubstituted homogalacturonic acid, and branched HG contains linear HG with a side chain consisting of α-1,4-d-galactosamic acid linked at position 2 or 3 of the main-chain Galpa residue. Up to now, we have studied the structure of purslane polysaccharides in many experiments. In order to understand the structure of purslane polysaccharides, we need further study. The structural characteristics of purslane polysaccharides are shown in Table 2.

## 4. Biological Activities of Purslane Polysaccharides

**Figure 3:**
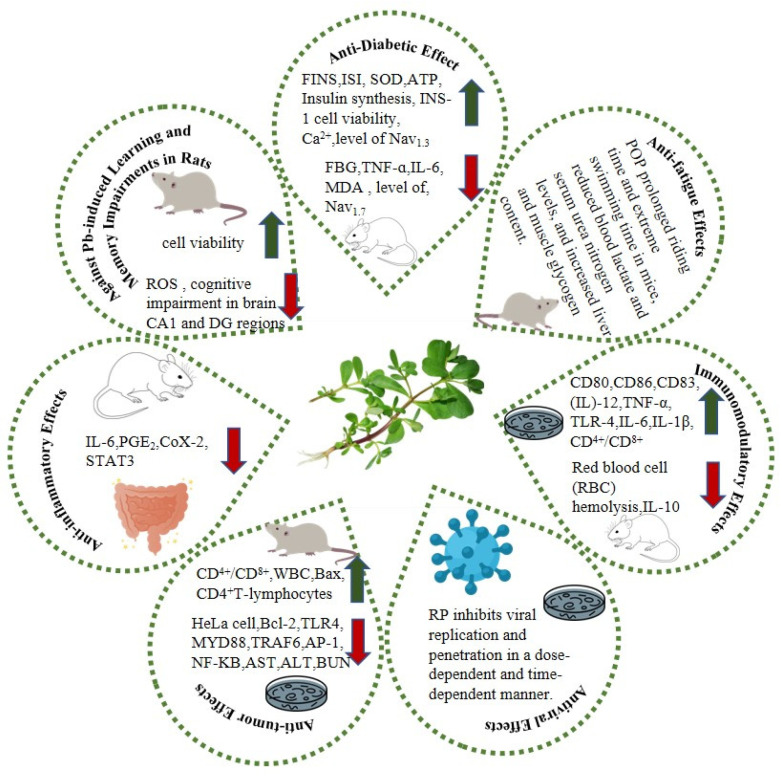
Biological activities of purslane polysaccharides (the red arrow indicates a downward revision; the green arrow indicates an upward revision. RP: pectin polysaccharide; POP: polysaccharides of purslane).

Purslane has been shown to be a natural plant with many health-promoting biological and pharmacological activities, and polysaccharides are an important component supporting these properties. The biological activity and health benefits of purslane polysaccharides have been extensively investigated both in vitro and in vivo. Table 3 contains comprehensive information about the biological activity of purslane polysaccharides. These polysaccharides demonstrate diverse effects, such as antifatigue, antidiabetic, antiviral, antitumor, anticolitis and immunomodulatory effects, as well as anti-lead poisoning (Figure 3).

### 4.1. Antifatigue Effects

It is well known that moderate exercise helps people to stay healthy and reduce mental stress, but excessive exercise can also lead to stress, fatigue and various other physical injuries [40]. There are few drug options available in modern medicine to treat fatigue, and some studies have shown that supplementation with natural substances can reduce exercise-induced physical fatigue and increase the physiological capacity of animals [41,42,43]. Therefore, natural medicines may have potential for research into antifatigue effects, and several studies have experimentally demonstrated the efficacy of purslane (POP) polysaccharide in reducing fatigue. In order to evaluate and verify the antifatigue performance of polysaccharides (POP) isolated from purslane, a spin test and a forced swim test were performed on male Kunming mice (18–22 g/animal) [44]. The results showed that the accumulation of blood lactic acid (BLA) during intense exercise may change the internal pH, causing so-called acidosis and leading to fatigue. Compared to the normal control group (NC), mice in the low-dose (LP), medium-dose (MP) and high-dose (HP) purslane polysaccharide POP supplementation groups showed significantly lower levels of BLA (*p* < 0.05), with BLA levels decreasing by 29.87%, 46.15% and 74.07%, respectively. The results indicated that POP was effective in delaying and reducing the production of BLA, thus delaying the onset of fatigue. In addition, serum urea nitrogen (SUN) is the result of protein and amino acid metabolism, which is another sensitive indicator of fatigue status. After POP treatment, compared with the NC group, the mice in the LP, MP and HP groups showed a significant decrease in SUN levels (*p* < 0.05) The decrease in SUN levels reflected a decrease in protein metabolism, which indicated an increase in endurance. Furthermore, compared to the NC group, mice in the LP, MP and HP groups showed significant increases in liver glycogen content (27.88%, 39.56% and 57.94%, respectively) and muscle glycogen content (24.41%, 38.97% and 44.61%, respectively) (*p* < 0.05), suggesting that the antifatigue activity of POP may be related to improved metabolic control of exercise and activation of energy metabolism. These results indicate that POP increased the amount of glycogen in the liver and muscle, while decreasing the levels of lactate and serum urea nitrogen in the blood, and also prolonged the duration of nonstop swimming and riding in mice [17]. In conclusion, purslane polysaccharides can have a potent antifatigue effect, providing an important natural medicine option for treating fatigue.

### 4.2. Antidiabetic Effect

Diabetes is divided into type 1 diabetes (T1D) and type 2 diabetes (T2D), but more than 90% of people with diabetes have type II diabetes [45,46,47]. Compared to the synthetic drugs currently used to treat diabetes, traditional Chinese medicine not only lowers blood sugar levels and effectively prevents or delays some of the long-term problems caused by diabetes, but also has fewer toxic side effects [48]. The crude water-soluble polysaccharide CPOP derived from purslane has demonstrated a noteworthy antidiabetic effect. Firstly, the newly prepared STZ solution 60 mg/kg was injected intraperitoneally into rats, and the same amount of citrate buffer was injected into the normal control group (NC). After 72 h, blood was taken from the tail bleeding, and the fasting blood glucose level was measured. The rats with a fasting blood glucose level of 11.1 mM were selected as the diabetic model. Subsequently, the effects of CPOP on body weight, glucose tolerance test (GTT), fasting blood glucose (FBG), fasting serum insulin (FINS) and insulin sensitivity index (ISI) were evaluated. The results showed that the body weight of diabetic rats increased significantly, glucose tolerance improved significantly, FBG decreased, FINS increased and ISI increased. At present, many studies have shown that the pathogenesis of type II diabetes may be related to cytokine-mediated inflammation. By observing the effects of CPOP on interleukin-6 (IL-6) and tumor necrosis factor-α (TNF-α) in model rats, it was shown that the levels of TNF-α and IL-6 in diabetic rats were significantly decreased after oral administration of 100, 200 and 400 mg/kg CPOP for 28 days (*p* < 0.05 or *p* < 0.01), indicating that CPOP may play an antidiabetic role through anti-inflammation. Furthermore, research suggests that reactive oxygen species (ROS) play a role in the development of diabetes and its complications, and the interconnection between diabetes and oxidative stress is well established. Malonaldehyde (MDA) is an important lipid peroxide, and a sensitive index to measure the level of free radical metabolism. After 28 days of intragastric injection of 100, 200 and 400 mg/kg CPOP in diabetic rats, it was found that compared with the model (MC) group, the MDA content of 100-CPOP, 200-CPOP and 400-CPOP groups was lower, and the SOD activity was higher. A significant statistical difference (*p* < 0.01) was observed in the levels of MDA content and SOD activity between the MC group and the CPOP group, suggesting that CPOP may exert an antidiabetic effect by mitigating oxidative stress [24].

**Figure 4:**
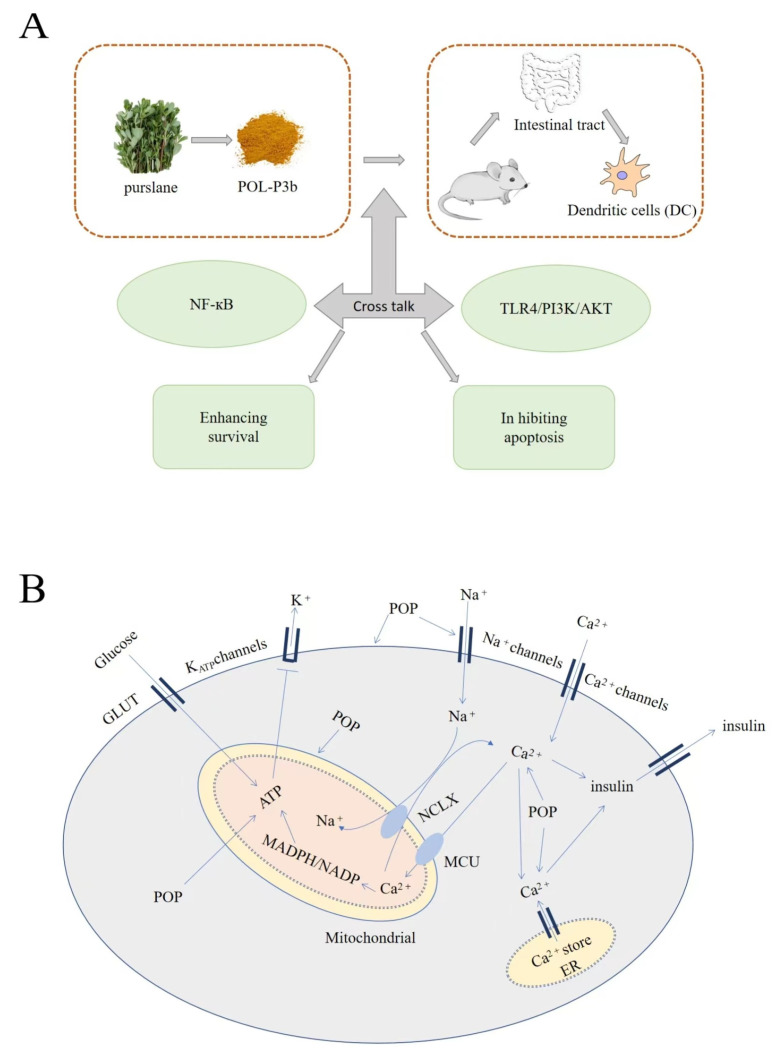
Mechanism diagram of purslane polysaccharides: (**A**) antitumor mechanism of purslane polysaccharides; (**B**) antidiabetic mechanism of purslane polysaccharides.

Another study showed that Purslane polysaccharides (POP) also have antidiabetic effects (Figure 4). The INS-1 cells’ viability was determined by 3-(4,5-dimethylthiazol-2-yl) 2,5-diphenyltetrazolium (MTT) assay [49]. INS-1 cells were seeded in culture dishes for 24 h. The medium was then removed and replaced with fresh medium containing POP (0.01–5 mg/mL) or TTX (0.1–1 μM). Insulin concentrations in culture medium and cell protein extracts were then determined. ATP Assay kit and photometer were used to determine ATP in cell lysate, and then the amount of ATP was determined by standard ATP curve. Glucose-stimulated insulin secretion (GSIS), intracellular Ca^2+^, mitochondrial membrane potential and membrane potential (MP) were measured in INS-1 cells. The results showed that POP and TTX (tetrodotoxin, a VGSC specific blocker) increased the concentration of insulin in the culture medium and the concentration of insulin in INS-1 cells under different concentrations of glucose. However, the effect of POP on insulin secretion and production was blocked by VGSC blocker TTX. POP also increased mitochondrial membrane potential, adenosine triphosphate (ATP) production, depolarization MP and intracellular Ca^2+^ level. In addition, in order to determine whether POP regulates the expression of VGSC, the expression levels of VGSC subunits NaV1.3 and NaV1.7 in INS-1 cells were detected. Western blotting analysis showed that POP treatment increased the expression level of Nav1.3 and decreased the expression level of Nav1.7. The above results indicate that POP may regulate insulin secretion through VGSC, and can also change intracellular Ca^2+^ release, ATP metabolism, cell membrane and mitochondrial membrane potential [26]. Based on the above studies, it can be speculated that purslane polysaccharides can be used as antidiabetic therapeutic agents. Although the research on their antidiabetic mechanism is limited, it can be inferred from the above studies that purslane polysaccharides have an antidiabetic effect and show good therapeutic effect. Therefore, they have the potential to develop new antidiabetic drugs with low toxicity.

### 4.3. Antiviral Effects

Viral infections remain a major source of illness and death around the world, with influenza, for example, causing some 3 million new cases and about one-tenth to one-sixth of people to die. Although the commonly used antiviral drugs have certain curative effects, they often cause serious adverse reactions [50,51]. Herbal extracts have a long history of therapeutic use, and some also have antiviral properties and are virtually free of toxic side effects [52,53]. The first reported isolation of antiviral polysaccharides from purslane included a neutral polysaccharide (RN), an acidic polysaccharide (RA) and a pectin polysaccharide (RP), and their antiviral characteristics against influenza A virus (IFV-A) and herpes simplex virus type 2 (HSV-2) were tested. When these three polysaccharides were added to Vero cells and Madin–Darby canine kidney (MDCK) cells infected with the virus, RP had the best inhibitory effect on virus replication during the subsequent culture, while the other two polysaccharides had little antiviral effect. A relatively high antiviral effect could also be observed if polysaccharides were added only during viral infection. However, when polysaccharides were added to the medium at different times after infection, the antiviral effect of RP was poor. Furthermore, experimental findings demonstrated the dose-dependent and time-dependent inhibitory effects of RP on virus penetration, but no dose-dependent interference with virus adsorption was observed. By limiting virus penetration without affecting virus adsorption, the polysaccharide RP shows potential antiviral activity, and it can be speculated that the virus penetration step is at least one of the targets of antiviral RP. To investigate the contribution of specific groups in the antiviral effect of RP, deesterified RP (DRP) was prepared by removing the methyl and acetyl groups from the GalA residues. Subsequently, the same experiments were conducted to examine the role of these groups in the antiviral activity of RP. However, the antiviral activity of RP was lost after deesterification, suggesting that methylation and acetylation of GalA residues in polysaccharide RP may be the cause of antiviral activity [19,54]. In general, according to the current research, Purslane polysaccharides show good antiviral properties, which provides potential value for further research. This finding may provide new directions and ideas for the development of antiviral drugs with good efficacy and fewer side effects.

### 4.4. Antitumor Effects

At present, the conventional treatment of cancer is often accompanied by many adverse reactions. The antitumor effect of natural substances has aroused the interest of researchers. Polysaccharides extracted from purslane have been found to have antitumor effects [55,56,57]. The antitumor mechanisms of polysaccharides can be roughly divided into two categories: (1) polysaccharides directly inhibiting the proliferation of tumor cells; (2) polysaccharides inhibiting the proliferation of cancer cells by activating the human immune system [58]. Firstly, purslane polysaccharides can directly inhibit tumor proliferation or induce apoptosis to exert antitumor effects. TLR4 expression and its downstream signaling pathway were examined using Western blotting, while the levels of inflammatory mediators were measured using an enzyme-linked immunosorbent assay (ELISA) kit. The apoptotic effect of POL-P3b on rat hepatoma cells was evaluated using WST-8 and Hoechst/Propidium iodide (PI) methods, confirming its antitumor activity. The results showed that POL-P3b showed obvious antiproliferation and apoptosis-inducing effects in LPS-induced HeLa cells, and downregulated the downstream signaling pathway of TLR4. At concentrations of 100 and 200 µg/mL, POL-P3b showed the effect of inducing apoptosis, upregulating the Bax level of HeLa cells and downregulating the Bcl-2 protein level, indicating that POL-P3b induced apoptosis by regulating the Bcl-2 family. In HeLa cells, POL-P3b not only inhibited the protein expression levels of TLR4, MyD88, TRAF6, activator protein-1 (AP-1) and nuclear factor-kB (NF-kB) subunit P65, but also reduced the production of cytokines and other chemical factors [59]. In addition, POL-P3b had a positive inhibitory effect on hepatocellular carcinoma cells. In summary, it can be speculated that the target of POL-P3b may be TLR4 on HeLa cells. POL-P3b induces apoptosis by activating the TLR4/NF-kB pathway to exert antitumor effects. Furthermore, in vitro and in vivo studies have reported the anticancer efficacy and mechanism of POL-P3b, specifically against cervical cancer. In this study, U14 mouse cervical cancer cells were used for animal experiments, and the growth inhibition effect of POL-P3b on HeLa cells was determined by measuring the MTT dye absorbance of living cells. The results showed that POL-P3b inhibited the growth of cervical cancer cells in vitro and in vivo, and significantly inhibited the tumor growth of U14 mice. The mechanism was related to sub-G1 cell cycle arrest, DNA damage and apoptosis [18]. In addition, sulfated polysaccharides also make a great contribution to antitumor activity [36]. The effects of purslane polysaccharide (POP1) and its five sulfated derivatives (POP1-s1, POP1-s2, POP1-s3, POP1-s4 and POP1-s5) on the human hepatoma cell line (HepG2) and cervical cancer cell line (Hela) were reported. The in vitro inhibitory effects of sulfate derivatives on HepG2 cells and Hela cells were observed by MTT assay. The results showed that natural POP1 had no cytotoxicity to HepG2 cells in vitro, but all sulfated derivatives had cytotoxicity. POP1-s3 showed the most obvious inhibitory effect; in particular, at a concentration of 2000 μg/mL, its inhibitory activity was the highest, reaching 51.3 ± 1.3%, indicating that sulfate modification can enhance the cytotoxicity of POP1 to tumor cells. Flow cytometry studies showed that Hela cells treated with sulfated derivatives could mediate cell cycle arrest in the S phase [29].

Secondly, the antitumor effect of purslane polysaccharides is also related to the enhancement or activation of the immune system. A study reported that a DC vaccine was prepared with mouse 4T1 tumor cell antigen to evaluate the characteristics of POL-P3b in inducing bone-marrow-derived DC maturation and function in mice. Studies have shown that POL-P3b induces the activation and maturation of dendritic cells (DCs), and its mechanism may be related to the enhancement of specific antitumor immune responses involving the TLR4/MyD88/NF-κB signaling pathway. In vivo experiments with BALB/c female mice showed that Pol-P3b-treated antigen-stimulated DCs significantly inhibited tumor growth by inducing apoptosis and enhancing immune response. In addition, hematological and blood biochemical tests and histopathological analysis were performed to evaluate the safety of POL-P3b. The results showed that the experimental mice were in good health and showed no signs of poisoning or death. It can be inferred that the toxicity of POL-P3b is very low, indicating that POL-P3b can be used as a new type of immune adjuvant for anticancer [38]. In addition, unlike injectable adjuvants, oral polysaccharides also have immunoregulatory antitumor effects. Firstly, the mouse cervical cancer model was established by s.c. injection of U14 cells. After 12 days of oral administration of POL-P3b in mice, it was found that the survival rate of intestinal DCs in mice was greatly increased, and its antitumor activity mechanism promoted the proliferation of intestinal DCs to inhibit the apoptosis of tumor cells (Figure 4). Speculatively, the mechanism by which oral administration of POL-P3b inhibits cervical cancer involves the induction of protection for intestinal dendritic cells (DCs) against tumor-induced apoptosis through activation of the TLR4/PI3K/AKT/NF-κB signaling pathway [37]. It is worth noting that another purslane polysaccharide (POP) has also been reported to have antitumor effects in vivo models. The activity of ICR mice sarcoma 180 tumor cells was determined. Experimental studies have demonstrated that POP exhibits notable inhibitory effects on the growth of transplantable sarcoma and enhances the immune response in animals. This includes increasing the count of white blood cells (WBC) and CD4+ T lymphocytes, as well as elevating the CD4+/CD8+ ratio. In addition, oral administration of POP also significantly increases the number of peripheral white blood cells and decreases serum AST, ALT, BUN and creatinine levels in tumor mice [22,60]. In short, purslane polysaccharides are natural product, with low toxicity and good biological safety. It can be used as a raw material or drug carrier for the development of new antitumor drugs, providing new ideas and choices for the development of antitumor drugs.

### 4.5. Anticolitis Effects

Colitis is an inflammatory bowel disease (IBD) characterized by symptoms such as bloody diarrhea and potential life-threatening complications. Research has indicated that the development and progression of IBD are closely linked to the dysregulation of inflammatory cytokines [61,62,63]. In order to prove the anticolitis effect of the purslane polysaccharide POLP, Wang et al. established an anticolitis model in mice. Mice weighing 20 ± 2 g were treated with ultrafiltrated water and standard food under standard laboratory conditions. At the same time, mice in the control group were given distilled water, and colitis mice were free to drink 5% dextran sulfate sodium (DSS) solution for 7 days.

The colitis-induced animals were randomly divided into four groups: a positive control group treated with sulfasalazine (9 mg/mL) and three groups treated with different concentrations of POLP (0.75, 0.5, 0.25 g/mL). The degree of inflammation and therapeutic effect of colitis were evaluated by clinical activity score (CAS) and H & E staining in histopathology. The results showed that the health status of colitis in the positive control group and the POLP group was significantly improved after 7 days of treatment. The CAS of the POLP group, particularly at a concentration of 0.75 g/mL, was comparable to that of the positive control group. Therefore, POLP can improve the health status of colitis mice. In addition, ELISA kits were used to measure the concentrations of four cytokines: IL-10, IL-1b, IL-6 and PGE2. The results showed that after 7 days of POLP treatment, the concentrations of IL-6 and PGE2 in the colitis group were significantly lower than those in the model group, but only IL-6 and PGE2 decreased. These findings suggest a close correlation between the therapeutic effect of POLP and the levels of these two cytokines. It has been observed that IL-6 can induce the phosphorylation of STAT3, and the upregulation of phosphorylated STAT3 is implicated in the pathogenesis of colitis, emphasizing the critical role of STAT3 in colitis development [64]. After POLP treatment, the phosphorylation level of STAT3 decreased sharply, indicating that POLP exerted its protective effect by regulating the phosphorylation level of STAT3. COX-2 can catalyze the synthesis of PGE2 from arachidonic acid. PGE2 is an important inflammatory cytokine that binds to G protein-coupled receptors to regulate inflammation and plays an important role in the development of colitis [65]. Considering that the synthesis of PGE2 is mediated by COX-2 and the phosphorylation of STAT3 can upregulate COX-2 expression, it is plausible to speculate that STAT3 serves as a pivotal protein in mediating the activity of POLP in colitis. Furthermore, previous reports have indicated that IL-6 can activate COX-2 through cross-signaling pathways. The IL-6-STAT3-COX-2 axis plays an important role in inflammatory diseases, and the expression level of COX-2 was also examined. Immunohistochemistry and Western blot analysis showed that the levels of STAT3 phosphorylation and COX-2 protein expression in the colitis group were significantly increased, and the levels of these two proteins were decreased after POLP treatment. These findings suggest a close association between the protective effect of POLP and the activation of STAT3 and COX-2. In conclusion, POLP can exert its anti-inflammatory effect by regulating the IL6/STAT3/COX-2 pathway and can effectively treat colitis [23]. Moreover, purslane polysaccharides have a history of more than 1000 years of clinical application in the treatment of colitis, suggesting that they may become a potential new treatment method. In the future, we can understand the mechanism of purslane polysaccharides more deeply and further explore their clinical value in the treatment of colitis. This may provide a more effective and safer treatment option for patients and open new research areas for the medical community.

### 4.6. Immunomodulatory Effects

The immune system is the body’s defense system, which performs immune response, immune function and self-protection [66]. It is composed of immune organs, immune cells and immune molecules. Natural compounds with immunomodulatory functions include polysaccharides, which can be extracted from some natural plants [67,68,69]. According to early studies, the purslane polysaccharide POL-P3b has an immunomodulatory function and can improve immunity. A study investigated the impact of POL-P3b on the maturation and functionality of mouse bone-marrow-derived DCs and explored the underlying mechanisms. Flow cytometry analysis confirmed the phenotypic maturation of DCs, revealing that POL-P3b upregulated the expression of CD80, CD86, CD83, and major histocompatibility complex class II molecules in DCs. Additionally, it stimulated the production of interleukin (IL)-12 and tumor necrosis factor-a, while reducing IL-10 levels. Furthermore, POL-P3b enhanced T cell proliferation driven by DCs and promoted apoptosis of U14 cells. Moreover, treatment with POL-P3b significantly upregulated the expression of TLR-4 on DCs. It can be speculated that POL-P3b may induce the maturation of DCs through TLR-4, and may be important for the molecular mechanism of immune enhancement, indicating that POL-P3b has a good immune enhancement effect [39]. In addition, PSPO also has immunomodulatory effects after successful selenization using Na_2_SeO_3_-HNO_3_ technology [70,71]. Two selenization products, SePSPO-1 and SePSPO-2, were produced after selenization, containing 753.8 and 1325.1 mg/kg selenium, respectively, while PSPO contained only about 80.6 mg/kg selenium. By using mouse spleen cells and RAW 264.7 macrophages as cell models, the results showed that SePSPO-1 and SePSPO-2 had greater immunomodulatory effects than PSPO (*p* < 0.05). In particular, the activity of SePSPO-1 and SePSPO-2 in macrophages was higher than that of PSPO, resulting in increased cell proliferation, increased macrophage phagocytosis and increased secretion of three immune-related cytokines, including tumor necrosis factor-α (TNF-α), interleukin-6 (IL-6) and IL-1β. On the other hand, SePSPO-1 and SePSPO-2 were more effective than PSPO in promoting interferon secretion, inhibiting the production of IL-4 by concanavalin A-or lipopolysaccharide-stimulated splenocytes or increasing the ratio of T helper cells (CD4+) to T cytotoxic cells (CD8+) in T lymphocytes. In general, the higher the degree of selenization of PSPO, the stronger the immunomodulatory effect on model cells; and the higher the polysaccharide dose, the greater the regulatory effect [20]. Therefore, it can be inferred that chemical selenization can be applied to the chemical modification of purslane polysaccharides to give them better immunomodulatory effects on immune cells.

In addition, another study reported that purslane polysaccharides can enhance immunity by scavenging free radicals. By inducing ovarian cancer in rats, the rats were divided into a control group and four purslane polysaccharide treatment groups (each group containing eight animals), and then the lymphocytes and thymus of the rats were removed and blood was collected for subsequent biochemical analysis. The results showed that the T lymphocyte stimulation index (SI) and B lymphocyte stimulation index (SI) of Wistar rats were significantly increased in a dose-dependent manner compared with the control group (*p* < 0.01). The inhibitory effect of purslane polysaccharides on erythrocyte hemolysis induced by a free radical initiator (H_2_O_2_) was dose-dependent. When the concentration was 1.2 mg/mL, the inhibition rate was the highest. Purslane polysaccharides also significantly promoted thymocyte proliferation (*p* < 0.01) in a concentration-dependent manner. In addition, polysaccharides from purslane had good superoxide anion, 1,1-diphenyl-2-picrylhydrazyl (DPPH), nitric oxide and hydroxyl radical scavenging activities, which were enhanced with the increase in extract concentration. The above results showed that purslane polysaccharides can scavenge excessive free radicals and enhance immunity [16]. In summary, these studies have shown that purslane polysaccharides have immunomodulatory effects, good effects and good development prospects.

### 4.7. Anti-Lead Poisoning Effects

Lead (Pb) is a widespread, nonbiodegradable and environmentally harmful metal that enters the environment through some human activities and can accumulate in the food chain [72,73]. The accumulation of lead (Pb) can endanger multiple physiological systems, including blood, kidney, bone and the central nervous system, and has a negative impact on people’s health [74,75]. Studies have reported that the purslane polysaccharide POP can inhibit lead-induced cognitive decline and has an anti-lead poisoning effect. In order to evaluate the protective effect of POP on lead (Pb)-induced neurotoxicity, in vitro and in vivo studies were conducted. Rat pheochromocytoma PC12 cells were cultured in DMEM containing 10% heat-inactivated fetal bovine serum and 1% penicillin–streptomycin solution, and then cultured in a humid environment of 95% air and 5% CO_2_ at 37 °C. MTT assay was used to determine cell viability. The results showed that the survival rate of PC12 cells exposed to lead increased after adding POP; the cell survival rate was the highest when 400 μg/mL POP was added; and POP was positively correlated with cell viability in a dose-dependent manner. It can be speculated that POP has a protective effect on Pb-induced PC12 cell injury. In order to understand whether POP can reduce lead-induced oxidative stress, the ROS level in PC12 cells was measured by DCFH-DA fluorescence. The results showed that the inhibitory effect gradually increased with the increase in POP concentration, and POP had an effective inhibitory effect on ROS production. In addition, in order to further study whether POP has a therapeutic effect on lead-induced cognitive dysfunction, rats were exposed to Pb for a long time and then supplemented with POP for 30 days. Morris water maze (MWM) analysis was performed at the end of supplementary administration to evaluate the learning and memory ability of rats. Compared with the Pb treatment group, the escape latency of rats in the 600 mg/kg/day POP group was significantly reduced from 47.38 s to 21.62 s, and the shorter escape latency indicated that their spatial learning and memory abilities were improved. Similar to the effect on escape latency, POP also prolonged the time to cross the platform. Two-way analysis of variance showed that the number of platform crossings gradually increased with the increase in POP doses from 200 to 600 mg/kg/day in Pb-treated rats, indicating that POP treatment alleviated the spatial learning and memory deficits of Pb-exposed rats. In addition, rats fed with 600 mg/kg/day POP recovered from Pb-induced dendritic spine loss. POP alleviated cognitive deficits in the CA1 and DG regions of the brain and significantly reversed the pb-induced dendritic spine defects in the CA1 and DG regions of the brain [15]. These findings suggest that purslane polysaccharide POP can reduce Pb-induced cognitive deficits and potentially prevent Pb-induced cognitive decline. In conclusion, purslane polysaccharide provides an important source of clues for anti-lead poisoning.

## 5. Structure–Activity Relationship and Structural Modification

### 5.1. Structure–Activity Relationship

Studies on purslane polysaccharide show that its good health benefits are closely related to its structure and conformation. At present, the research on the structure activity relationship of polysaccharides mainly includes molecular weight, monosaccharide composition, type of glycosidic bond, main chain, branch chain and spatial configuration. The monosaccharides of POP-L and CPOP obtained from purslane differ in composition, but they both have antidiabetic effects. The monosaccharide composition of POP-L is mainly composed of Rha, Ara, Glc and Gal, while CPOP is composed of Rha, Ara, Xyl, Man, Glu and Gal. The monosaccharides have the same composition and different proportions and different biological activities. POL-P3b and purslane polysaccharides are composed of Glu and Gal with molar ratios of 0.5:1.00 and 2.3:1.0, respectively. The former showed antiviral activity and the latter had an immunomodulatory effect. This is not the only study on the structure–activity relationship of polysaccharides. At present, the study on the structure–activity relationship of polysaccharides in purslane is still superficial. In order to improve the biological activity of polysaccharides, further research and exploration should be carried out in the future.

### 5.2. Structural Modification

Structural modification is a common method to enhance biological activity. Covalent selenization, one of the chemical modification techniques, is frequently used in polysaccharide molecular modification. The antitumor activity of PPS was studied by selenization modification. Compared with PPS, the selenium content of SePPS1 and SePPS2 increased by 8 and 26 times, respectively. In vitro experiments, human colon cancer HCT-116 cells were treated with different concentrations of PPS, SePPS1 and SePPS2 to observe their inhibitory activities on the cells. The results showed that PPS, SePPS1 and SePPS2 inhibited HCT-116 cells, and the inhibition rates were 14.2–24.4%, 15.2–29.8% and 17.2–43.4%, respectively. In addition, SePSPO-1 and SePSPO-2, the selenate products of purslane polysaccharides (PSPO), have been shown to have higher immunomodulatory effects in another study. In addition, another study showed that SePSPO-1 and SePSPO-2, the selenate products of PSPO, had higher immunomodulatory effects, and SePSPO-2 had higher degree of selenization than SePSPO-1. Mouse spleen cells and RAW264.7 macrophages were used as models to evaluate their immunomodulatory effects. The results showed that the cells treated with PSPO, SePSPO-1 and SePSPO-2 enhanced the secretion of IL-6, IL-1β and TNF-α, and SePSPO-2 enhanced the secretion of cytokines the most. Therefore, the higher the degree of selenization, the stronger the activity. In conclusion, the selenium polysaccharide of purslane can improve its antitumor and immunomodulatory effects.

Sulfation is also a common chemical modification method. Chen et al. modified POP by chlorosulfonic acid–pyridine method, and added N,N-Dicyclohexylcarbodiimide (DCC) to the reaction system as the dehydration condensation agent; four sulfated derivatives of POP1 (POP1-s1, POP1-s2, POP1-S3 and POP1-s4) were obtained. The average molecular weight (MW) of sulfonated derivatives ranged from 41.4 kDa to 48.5 kDa, and the degree of substitution (DS) ranged from 1.01 to 1.81. Two special absorption bands (1258 and 815 cm^−1^) were observed in the FT-IR spectra, indicating that the sulfation reaction took place. Preliminary in vitro experiments showed that all sulfate derivatives exhibited cytotoxicity to HepG2 cells, with POP1-s3 having the highest inhibitory activity. In addition, the experiments also showed that POP1 had lower inhibitory activity on Hela cells, while POP1-s1, POP1-s2 and POP1-S3 had higher inhibitory activity on Hela cells. Thus, sulfonated treatment of native POP1 can enhance its cytotoxicity against tumor cells, and sulfate derivatives of POP1 will play an auxiliary role alongside anticancer drugs in future cancer therapies.

## 6. Applications of Purslane Polysaccharides

### 6.1. In the Food Industry

With rising living standards and changing dietary trends, people are becoming more health-conscious, and greens are becoming popular with a wide range of consumers [76]. Purslane is rich in pectin polysaccharides and has good absorption properties [77]. Therefore, adding purslane to flour not only improves the softness of the dough but also increases the nutritional value of the bread. Using four different concentrations of purslane (0%, 5%, 10% and 15%) added to the flour, the absorption, mix stability and softness, as well as the protein, fat, moisture and fiber content of the bread, were investigated as the concentration of purslane flour increased. The results showed that the best prescription containing 10% purslane flour had the best acceptability in terms of sensory characteristics, color, flavor, texture and overall preference. The addition of purslane to bread not only improves the taste of bread, but also has health benefits. It is well known that the sensory perception of consumers is crucial for the distribution of products on the market. Purslane as a food product can not only improve the nutritional and bioactive composition of the product, but can also influence its sensory perception and processing properties. Therefore, it is worthwhile to try the addition of purslane to the development of new bread products. In addition, purslane polysaccharides as a thickening agent can be added to yoghurt as a stabilizer, not only to enhance its gel network structure, effectively reduce whey precipitation, improve gel texture and enhance yoghurt stability, but also to blend a unique and acceptable flavor [78]. As polysaccharides act as a carbohydrate, they can provide nutrients to the fermenting flora of yoghurt and participate in biochemical reactions as well as the synthesis of organic matter, increasing the different nutritional properties of yoghurt, such as improving immunity, regulating intestinal flora and being beneficial to human health. In addition, a health wine prepared by fermenting purslane with other herbs is rich in purslane polysaccharides, flavonoids and other nutritional components, with functions such as lowering blood sugar and improving immunity, as well as antibacterial and anti-inflammatory functions. The production process has been patented, and is in force (CN103642623A) [79]. Moreover, widely consumed as a wild vegetable in Europe, Africa and Asia, as well as in some parts of Australia, purslane has a slightly sour and savory flavor to its stems and leaves, and is commonly found in salads, soups and stews [80]. As an edible plant, purslane is highly nutritious, and can be used to clear heat and detoxify the body if simmered in a soup with green beans. As a result, research into its use as a functional food has recently attracted a lot of attention. Purslane is extremely abundant worldwide and is simple and inexpensive to grow. The research and market development of purslane is therefore very promising, and deserves to be studied in depth in the context of food industrialization.

### 6.2. In the Pharmaceutical Industry

Purslane contains a variety of chemical components such as flavonoids, organic acids, alkaloids, polysaccharides and terpenoids, making it a natural plant with a wide range of pharmacological activities. Pharmacological studies have shown that purslane has a variety of therapeutic effects, and is used clinically for antibacterial, anti-inflammatory and free-radical-scavenging and immune-boosting purposes [81,82]. In recent years, there has been increasing interest in the polysaccharides extracted from purslane. Pharmaceutical research on purslane polysaccharides has focused on the treatment of diabetes, as well as anti-inflammatory, antitumor, antiviral, antifatigue, anti-lead poisoning and immunomodulation effects. Because of its abundance, safety, and low toxicity, it has great potential and value for exploitation. In China, purslane is an important part of traditional Chinese medicine formulations with immunosuppressive and viral suppressive effects. For example, purslane soup, which uses purslane as major component, is mainly used for the treatment of bacillary dysentery and enteritis, which may be related to the anti-inflammatory and immunomodulatory effects of purslane polysaccharides. Purslane is also used in combination with other herbs for the treatment of bowel cancer, where the antitumor effects of purslane polysaccharides may play a role in the treatment. It is also used as an anti-inflammatory and antibacterial agent by decocting fresh purslane in water or wine and pounding it externally, or by taking its juice and using it in water. Purslane polysaccharides have anti-inflammatory properties, and it can be assumed that the polysaccharides play their corresponding role in this process. In addition to this, in the field of pharmaceutical technology, one patent discloses the use of a purslane polysaccharide in the preparation of an antiacute lung injury drug. Some studies have also shown that purslane polysaccharide (POL-P3b) can be used as a novel immune adjuvant in the treatment of breast cancer. Moreover, purslane polysaccharides can be used as an edible adjuvant or nutraceutical to boost the immune system for antitumor, hypoglycemic and immune-boosting effects. However, further research is needed to determine the mechanism of action of purslane polysaccharides in specific foods and the anticancer activity in beverages to protect the health interests of consumers. In conclusion, with the continuous development of science and technology, the mechanism of action of purslane will be further studied and elucidated, which will lead to better pharmaceutical applications and further contribution to the human treatment of chronic diseases such as tumors and diabetes.

### 6.3. In the Cosmetics Industry

Many natural plants are not only used as medicine, but can also be added to cosmetics for their effects [83]. With the in-depth study of purslane polysaccharides, it was found that they also have a high value and potential for use in everyday cosmetics. Purslane polysaccharides have good antiallergic, antibacterial and anti-inflammatory effects against various external skin irritations, and are also effective against acne, eczema, dermatitis and itchy skin [84]. According to incomplete statistics, an increasing number of people worldwide consider themselves to have sensitive skin, 38% of whom are men and 62% of whom are women. Cosmetics with antiallergic properties are therefore becoming increasingly important to consumer choice, and cosmetic ingredients with antiallergic activity, especially those of natural plant origin, are becoming more popular in the market [85,86]. Several studies have shown that purslane is effective in treating septic skin problems such as acne and pimples, and these effects are inextricably linked to the ingredient purslane polysaccharide. Purslane polysaccharides reduce the secretion of the inflammatory factor interleukin, which has some anti-inflammatory effects, thus soothing skin inflammation, which is the cause of acne pimples [87]. This has created a vast market and substantial economic benefits for the cosmetics industry, driven by consumer demand for natural plant actives, and purslane polysaccharides meet the consumer demand for a nontoxic source of product ingredients for the human body. This also suggests that the cosmetic industry should actively explore new and reliable natural plant-based skin care ingredients to improve the safety performance of their products. Creams containing purslane extract have been reported to improve dry, rough, red and flaky skin [88]. Based on the structural characteristics of polysaccharides, which have excellent moisturizing properties, it can be assumed that it is likely that the polysaccharides play a corresponding role. There are thousands of cosmetic products that use purslane extract as a main efficacious ingredient, such as Winona, MUJI and Bacitracin. However, the extent to which purslane polysaccharides can play a positive role in cosmetics is yet to be proven. There is still a long way to go when it comes to cosmetics based on polysaccharide molecules rather than aqueous extracts. In subsequent studies, the constitutive relationship, purification process, onset of action mechanism, penetration mechanism and processing compounding of purslane polysaccharides should be further explored in depth. It is believed that in the near future, purslane polysaccharide cosmetics will play a more important role in the field of skin care and cosmetics, and contribute to meeting the needs of consumers.

### 6.4. In the Livestock Industry

**Figure 5:**
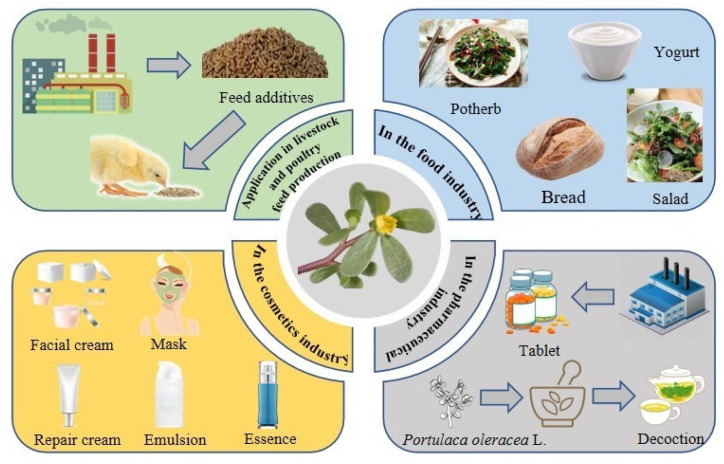
Practical applications of purslane polysaccharide.

Due to the various pharmacological effects and fewer toxic negative effects of plant polysaccharides, polysaccharides have also been widely used in animal husbandry, such as Astragalus polysaccharides and Oxalis polysaccharides, both of which have been used to good effect [89,90]. Similarly, purslane polysaccharides can also have corresponding applications in animal husbandry. Ge Jian et al. showed that purslane polysaccharides could significantly increase the indices of the thymus, spleen and bursa of chicks, and significantly increase the levels of SOD, GSH-Px, CAT and T-AOC in chicks. The results showed that the cell proliferation index was mainly in the G1/G0 phase, with a few cells in the S and G2/M phases, and the cell cycles of different immune organs were similar. This indicates that purslane polysaccharides can promote the growth and development of chicken immune organs and the cell proliferation of chicken immune organs, enhance the immune function and improve the immunity of the organism, which is obviously a better alternative to the common feed. In addition, Zhang Ting et al. showed that horsetail polysaccharide has some protective effect on pig sperm. For example, the addition of 0.25 mg/mL of purslane polysaccharides to the traditional diluent TCG during the freezing of pig semen improved the viability, survival, plasma membrane integrity, acrosome integrity and mitochondrial activity of pig sperm after freezing. Therefore, the addition of purslane polysaccharides to feeds can be used to a certain extent as a substitute for antibiotics without affecting animal performance in order to improve animal food quality and meat safety. Although there are some preliminary studies on purslane polysaccharides in animal husbandry, there are still many problems to be solved, and further in-depth studies are needed. It is believed that with the improvement of the polysaccharide extraction process and further research, the application potential of purslane polysaccharides in livestock and poultry feed production will be further developed, and in the future, feed containing purslane polysaccharides may be mass-produced and used on a large scale (Figure 5).

In summary, purslane polysaccharides are an important material base component for purslane to play pharmacological and health functions. They are widely used and involve many fields. Therefore, the research and development work of purslane polysaccharides in various industries must be continuously and deeply carried out, especially in the field of food, so as to contribute to the extension of the deep food processing industry chain and the sustainable development of food and medicinal industry.

## 7. Conclusions and Perspectives

Due to the increasing emphasis on dietary and physical health among people, purslane, as a natural plant with edible, medicinal and nutritional value, has attracted much attention. Among them, purslane polysaccharides have attracted research interests in many fields, such as food, pharmacy, cosmetics, and animal husbandry, due to their wide range of sources and varieties, as well as their antidiabetic, anti-fatigue, anti-lead poisoning, immunity-enhancing, anti-inflammatory, antitumor, antiviral, antioxidant and other effects. Purslane polysaccharides have made certain progress in extraction and purification, structural characteristics, health benefits and other aspects. In order to facilitate future research, this review has organized purslane polysaccharides from these aspects mentioned above and summarized their existing and potential applications.

As one of the most important steps in obtaining polysaccharides, extraction often affects the yield, chemical structure, quality and biological activity of polysaccharides. Currently, most polysaccharides from purslane are extracted through hot water extraction. With the continuous progress and development of technology, various methods such as microwave, ultrasound and enzymatic methods have been used to extract polysaccharides, promoting the improvement of the polysaccharide extraction rate and enabling more bioactive polysaccharides to be obtained. The microwave-assisted extraction method has strong transmissibility. During the extraction process, the microwave can penetrate the plant histiocyte to disperse the polysaccharide into the external solvent. This extraction method has the characteristics of environmental friendliness and the low use of organic reagents. Applying the microwave-assisted extraction method to extract polysaccharides from purslane can solve the problem of residual organic reagents in edible polysaccharides, while also avoiding the weakening of polysaccharide activity by high temperature. Ultrasound-assisted extraction is a method that utilizes multiple effects, such as vibration, stirring, cavitation, infiltration and penetration, to act on cellular contents. For purslane, a thick and succulent plant, ultrasound can be used to better dissolve polysaccharides. Each extraction method has both advantages and disadvantages, and several methods can be combined to maximize the advantages of each method. In actual production, a suitable extraction method and optimal extraction process for purslane polysaccharides can be selected based on comprehensive considerations such as extraction rate, production cost and ease of operation. The existing research provides a valuable reference for optimizing the extraction conditions of purslane polysaccharides.

The analysis of the primary structure of purslane polysaccharides is a prerequisite for the in-depth exploration of their activity and application. At present, the analysis of the primary structure of purslane polysaccharides is relatively mature, and there is increasing research on their monosaccharide composition and relative molecular weight. The primary structure is the foundation of the advanced structure. However, the chemical structure of purslane polysaccharides is complex, and there are relatively few reports on their advanced structure and spatial conformation. The gaps and difficulties in this part of the content need to be addressed in the next step. Exploring the structure–activity relationship is a good solution to this difficult problem. Clarifying the relationship between structure and activity is helpful for studying the structure of purslane polysaccharides. On the other hand, it is beneficial for improving or enhancing biological activity by changing the structure so as to maximize the utilization of purslane polysaccharides.

Numerous studies have shown that purslane polysaccharides have multiple health benefits, and this has been confirmed through numerous in vitro and in vivo experiments. Among them, purslane polysaccharides have good antifatigue effects, and strengthening research on their antifatigue effects can provide ideas for the future development of new antifatigue health products. However, the pharmacological mechanism of its action has not been fully elucidated. The existing models have single evaluation indicators and cannot reflect fatigue caused by multiple factors. To elucidate the antifatigue mechanism of purslane polysaccharides, a more representative and applicable pharmacological model is needed to specifically study the biological activity of purslane polysaccharides. In addition, most people are currently in a long-term state of subhealth. Among them, fatigue is the main manifestation of subhealth, and it has become an important factor affecting people’s quality of life. Purslane polysaccharides exhibit good antifatigue effects, and increasing research on their antifatigue effects can provide ideas for the development of new anti-fatigue health products in the future. Regarding drug development, chemical modification provides ideas, as it can significantly increase structural diversity and enhance activity. For example, covalent chemical selenization enhances the immune regulation and antitumor effects of purslane polysaccharides, while sulfation modification enhances the cytotoxicity of purslane to tumor cells. Therefore, the chemical modification of polysaccharides is an effective method to enhance their activity. With the improvement of modification methods and in the technological level, polysaccharide derivatives will have an increasingly broad development space.

Although purslane polysaccharides, as natural plant polysaccharides, are nontoxic and have high affinity for the human body. There is a lack of systematic and reliable quality control standards for purslane polysaccharides to be better applied to the protection and treatment of human diseases. Therefore, the quality evaluation research of purslane polysaccharides and their related products is crucial to ensure the effectiveness and safety of the products. It is also essential to control the quality of purslane polysaccharides through quantitative and qualitative analysis.

Purslane, as an edible resource and high-quality medicinal, has broad application prospects in multiple fields due to its polysaccharide components. Therefore, the extraction and purification of polysaccharides from purslane, the study of chemical structure and activity, and further exploration of structure–activity relationships are of great significance. With the deepening of research, purslane polysaccharides will gradually become an important component of people’s daily life, helping people to have a healthy diet, supplement nutrients and prevent diseases.

## Figures and Tables

**Table 1:** A summary of purslane polysaccharide extraction and purification methods.

Extraction					Purification			Ref.
Polysaccharide Fraction	Extraction Methods	Time (h/min)	Temperature (°C)	Solid–Liquid Ratio	Total Yield (%)	Polysaccharide Fraction	Purification Methods	
POP-T	Hot water extraction	6 h	100 °C	1:20	4.84%	POP-T	Sevag method and dialysis	[15]
Purslane polysaccharides	Hot water extraction	6 h	100 °C	1:8	7.00%	Purslane polysaccharides	HPLC	[16]
POP-X	Hot water extraction	9 h	100 °C	1:10	9.41%	POP-X	Filtering and centrifuging	[17]
POL-P	Hot water extraction	6 h	95 °C	1:4	N/A	POL-P3b	DEAE cellulose and Sephadex G-200 column chromatography	[18]
RH1 and RH2	Hot water extraction	3 h	N/A	69:122	0.43 × 10^−3^%	RN	Ion-exchange column chromatography and gel filtration	[19]
					0.10 × 10^−2^%	RA		
					0.35 × 10^−2^%	RP		
PSPO	Enzyme-assisted method	4 h	90 °C	1:20	N/A	PSPO	N/A	[20]
CPOPW	Hot water extraction	9 h	100 °C	4:25	N/A	POPW-HG	DEAE -cellulose column and Sepharose CL-6B gel column	[21]
DCPOP	Hot water extraction	9 h	75 °C	1:40	0.90%	POP-S	DEAE- Cellulose anion-exchange chromatography and Sepharose CL-6B gel-permeation chromatography	[22]
POLP	Hot water extraction	12 h	100 °C	N/A	6.08%	POLP	Sevagmethod	[23]
CPOP	Hot water extraction	9 h	80 °C	1:20	N/A	N/A	N/A	[24]
POP-Z	UAE	53 min	61 °C	1:39	13.55%	POP-Z	Centrifugal	[25]
POP-H	Hot water extraction	4.5 h	90 °C	1:15	N/A	POP-H	DEAE cellulose, Sephadex G-200 column chromatography and dialysis	[26]
POP-Y	Double-enzyme extraction method	100 min	50 °C	1:25	19.83%	POP-Y	Centrifugal	[27]
POP-Su	Ultrasonic complex enzymatic method	51 min	N/A	1:31	21.23%	POP-Su	N/A	[28]
Crude POP	N/A				42.30%	POP1-C	Dialysis and SphadexG-100 column	[29]
	N/A				4.50%	POP2-C		
	N/A				6.20%	POP3-C		
POP-Ch	EAE	2 h	39 °C	1:20	4.22%	POP-Ch	N/A	[30]
POP-J	MAE	10 min	60 °C	1:30	13.87%	POP-J	N/A	[31]
POP-Xu	UHP	5 min	N/A	1:20	22.21%	POP-Xu	Sevag method	[32]

N/A means not mentioned.

**Table 2:** Compound name, molecular weight, monosaccharide composition, structure of purslane polysaccharides and analytical technique.

Compound Name	Molecular Weight	Monosaccharide Composition	Structure	Analytical Technique	Ref.
RN	8.3 kDa	Glu:Man:Arab:Gal = 0.1:38.8:13.7:5.3	RN contained 1,4-linked Manp, 1,4-linked Glcp and 1,2,4-linked Glcp residues with small amounts of 1,4,6-linked Manp, 1,4,6-linked Glcp and terminal-linked Galp residues, and Araf residue was suggested to be mainly present as nonreducing terminal-linked residues.	GC, HPLC	[19]
RA	58 kDa	Ara:Gal:Rha:Xyl:GlcA = 23.2:67.0:2.8:2.9:4.1	RA consists of a-1,3-linked *β*-d-Galp main chain, partially substituted at C-6 by 1,6-linked *β*-d-Galp side chains. Moreover, GlcA was present in RA in the forms of terminal-linked and 1,4-linked residues.		
RP	87 kDa	GalA:Gal:GlcA:Ara:Rha = 67.8:11.3:10.6:5.8:4.3	RP consists of a-1,4-linked GalA residues that are highly methylated and partially acetylated.		
POP1-C	55.8 kDa	N/A	N/A	FT-IR, NMR	[29]
Purslane polysaccharide	N/A	Glu:Gal = 2.3:1	N/A	HPLC, PDA	[16]
POP-T	1.55 × 10^4^ kDa	Gal:Ara:Rha = 50.92:32.32:16.75	There were hydroxyl groups, C-O, β-configurations and uronic acid in the structure of POP-T.	GC, HPGPC, FT-IR	[15]
POP-S	24.6 kDa	Man:Ara:Glu:Gal = 2.1:5.2:2.1:11.2	N/A	GC, HPGPC	[22]
POP-H	11 kDa	Man:Rha:GlcA:GalA:Glu:Gal:Ara = 5.3:5.9:1.3:27.6:1:18.8:14.6	N/A	HPLC, IR, HPGPC	[26]
POL-P3b	0.253688 kDa	Glu:Gal= 0.75:1.00	POL-P3b structure had hydroxyl groups and β-glucoside bonds.	HPLC, UV, IR	[18]
POP-L	8.03 kDa	Rha:Ara:Glc:Gal = 1:1.16:0.23:0.59	N/A	N/A	[35]
POPW-HG	41.2 kDa	Galacturonic acid = 95% A trace of Man and Rha	POPW-HG consists of 1, 4-GALA with hydroxyl, uronic acid and pyranose rings.	HPSEC, GC, NMR, FI-IR	[21]
CPOP	7.3 kDa, 11.9 kDa, 93 kDa	Rha:Ara:Xyl:Man:Glu:Gal = 1:1.1:1.3:1.9:2.4:3.4:1	N/A	GC, HPSEC	[24]

N/A means not mentioned.

**Table 3:** Biological activity of purslane polysaccharides and their underlying mechanisms of actions.

Biological Activity	Polysaccharide Name	In Vitro or In Vivo	Indicated Concentration	Models/Test System	Action or Mechanism	Ref.
Antifatigue effects	POP-X	In vivo	75, 150 and 300 mg/kg	Male KM mice	POP prolongs riding time and extreme swimming time in mice, reduces blood lactate and serum urea nitrogen levels and increases liver and muscle glycogen content.	[17]
Antidiabetic effects	POP-L	In vitro	0.5 mg/mL	INS-1 cells	POP increases mitochondrial membrane potential and ATP production; depolarises cell membrane potential (MP), intracellular Ca^2+^ levels ([Ca^2+^]) and Nav_1.3_ expression levels; and decreases Nav_1.7_ expression levels.	[26]
	CPOP	In vivo	100, 200 and 400 mg/kg	SD rats	CPOP appears to significantly reduce FBG, TNF-6, IL-6 and MDA levels and increase FINS, ISI and ROS levels in diabetic rats.	[24]
Antiviral effects	RP	In vitro	234 mg	Cells and Viruses V ero and Madin–Darby canine kidney (MDCK) cells were grown in minimal essential medium (MEM) containing 5% fetal bovine serum (FBS)	RP has been shown to exert potential anti-HSV-2 activity by inhibiting viral penetration without inhibiting viral adsorption.	[19]
Antitumor effects	POP1(POP1-s1, POP1-s2, POP1-s3 and POP1-s4)	In vitro	100–2000 μg/mL	HepG2 and Hela cells	Inhibits the growth of Hela cells in S-phase and induces apoptosis through cell cycle arrest.	[29]
	POL-P3b	In vitro	100 or 200 mg/mL	HeLa cells	POL-P3b induces apoptosis in HeLa cells by upregulating Bax levels and downregulating Bcl-2 protein levels, while inducing apoptosis in part by regulating the Bcl-2 family. The target of POL-P3b is probably TLR4 on HeLa cells, and POL-P3b induces apoptosis through activation of the TLR4/NF-kB pathway.	[36]
	POP-S	In vivo	25, 50, and 100 mg/kg	ICR mice	POP significantly inhibits the growth of transplantable sarcoma 180, increases the number of white blood cells (WBC) and CD4+ T lymphocytes and increases the CD4+/CD8+ ratio. In addition, oral administration of POP significantly increases the number of peripheral blood leukocytes and reduces serum AST, ALT, BUN and creatinine levels in tumor-bearing mice.	[22]
	POL-P3b	In vivo	50, 100 and 200 mg/kg	Female KM mice	Pol-p3b induces tumor-induced apoptosis in DC cells by stimulating the TLR4-PI3K/AKT-NF-κB signaling pathway.	[37]
	POL-P3b	In vivo	50 µg/mL and 100 mg/mL	BALB/c female mice	The mechanism of action may be related to the enhancement of specific antitumor immune responses involving the TLR4/MyD88/NF-κB signaling pathway.	[38]
	POL-P3b	In vitro and in vivo	250, 500 and 1000 μg/mL 50, 100 and 200 mg/kg	HeLa and U14 cells, Female KM mice	POL-P3b inhibits the growth of cervical cancer cells in vitro and in vivo, and also significantly inhibits tumor growth in U14 mice.	[18]
Anticolitis effects	POLP	In vivo	0.75, 0.5 and 0.25 g/mL	KM mice	POLP exerts its protective effect through regulation of the IL-6/STAT3/COX-2 pathway.	[23]
Immunomodulatory effects	PSPO (SePSPO-1, SePSPO-2)	In vitro	753.8 and 1325.1 mg/kg	Female BALB/c	The higher the degree of selenylation of PSPO, the stronger the immunomodulatory effect on model cells, the increased phagocytosis of macrophages and the increased secretion of cytokines related to immunity.	[20]
	POL-P3b	In vivo	250, 500 and 1000 μg/mL	Recombinant mouse GM-CSF, mouse CD11c, FITC antimouse CD80, FITC antimouse CD83, PE antimouse CD86 and PE antimouse MHC-II	The expression of TLR-4 is significantly increased in POL-P3b-treated dc, which may induce dc maturation through TLR-4, and this has important implications for the molecular mechanism of POL-P3b immune enhancement.	[39]
	Purslane polysaccharide	In vitro and in vivo	1, 5, 10, 20 and 40 μg/mL	Wistar rats; thymocytes	Purslane polysaccharides scavenge excess free radicals and boost the immune system.	[16]
Anti-lead poisoning effects	POP-T	In vitro and in vivo	600 mg/kg/day	PC12 cells and rats	POP is protective against pb-induced oxidative toxicity in PC12 cells by reducing ROS production and increasing cell viability and also attenuates cognitive deficits in brain CA1 and DG regions and significantly reverses pb-induced spinal deficits in brain CA1 and DG regions.	[15]

## Data Availability

Not applicable.
